# A schedule for tapering glucocorticoid treatment in patients with severe SARS-CoV 2 infection can prevent acute adrenal insufficiency in the geriatric population

**DOI:** 10.1007/s42000-024-00564-9

**Published:** 2024-05-17

**Authors:** Irene Tizianel, Elena Ruggiero, Marianna Torchio, Matteo Simonato, Chiara Seresin, Francesco Bigolin, Ilaria Pivetta Botta, Giulia Bano, Mario Rosario Lo Storto, Carla Scaroni, Filippo Ceccato

**Affiliations:** 1 Endocrine Unit-Department of Medicine DIMED, Via Ospedale Civile, Padova, 105 - 35128 Italy; 2 Endocrine Unit, University Hospital of Padova, Padova, Italy; 3 Geriatric Division, University Hospital of Padova, Padova, Italy; 4 Pain Therapy and Palliative Care, Veneto Institute of Oncology IOV–IRCCS, Padua, Italy

**Keywords:** COVID-19, Glucocorticoid therapy, Adrenal insufficiency

## Abstract

**Objective and design:**

Glucocorticoids (GCs) have been widely used in symptomatic patients for the treatment of COVID-19. The risk for adrenal insufficiency must be considered after GC withdrawal given that it is a life-threatening condition if left unrecognized and untreated. Our study aimed to diagnose adrenal insufficiency early on through a GC reduction schedule in patients with COVID-19 infection.

**Patients and measurements:**

From November 2021 to May 2022, 233 patients were admitted to the Geriatric Division of the University Hospital of Padova with COVID-19 infection. A total of 122 patients were treated with dexamethasone, after which the GC tapering was performed according to a structured schedule. It consists of step-by-step GC tapering with prednisone, from 25 mg to 2.5 mg over 2 weeks. Morning serum sodium, potassium, and cortisol levels were assessed 3 days after the last dose of prednisone.

**Results:**

At the end of GC withdrawal, no adrenal crisis or signs/symptoms of acute adrenal insufficiency were reported. Median serum cortisol, sodium, and potassium levels after GC discontinuation were, respectively, 427 nmol/L, 140 nmol/L, and 4 nmol/L (interquartile range 395–479, 138–142, and 3.7–4.3). A morning serum cortisol level below the selected threshold of 270 nmol/L was observed in two asymptomatic cases (respectively, 173 and 239 nmol/L, reference range 138–690 nmol/L). Mild hyponatremia (serum sodium 132 to 134 nmol/L, reference range 135–145 nmol/L) was detected in five patients, without being related to cortisol levels.

**Conclusions:**

A structured schedule for the tapering of GC treatment used in patients with severe COVID-19 can reduce the risk of adrenal crisis and acute adrenal insufficiency.

## Introduction

The clinical spectrum of coronavirus disease 2019 (COVID-19) is heterogeneous, ranging from asymptomatic carriers to patients with severe progressive pneumonia requiring hospitalization or intensive care [1, 2].

Glucocorticoids (GCs), alone or combined with other drugs, are widely used in symptomatic patients for treatment of COVID-19. The RECOVERY trial, published in July 2020, reported that high doses of dexamethasone (6 mg) for a brief period (up to 10 days) are effective in reducing 28-day mortality among patients who were receiving either invasive mechanical ventilation or oxygen alone [3]. However, dexamethasone is the most potent synthetic GC, with 1 mg being able to suppress hypothalamic-pituitary-adrenal (HPA) axis for at least 12 h [4]. Therefore, adrenal insufficiency must be considered after GC withdrawal given that the latter is a life-threatening condition if left unrecognized and untreated.

Central adrenal insufficiency (CAI) is characterized by reduced adrenal cortisol production in response to inappropriate adrenocorticotropin (ACTH) secretion as a result of endogenous hypothalamic-pituitary injury or exogenous HPA axis suppression due to GC administration [5, 6]. GC treatment is largely used in the general population (up to 2%): central adrenal insufficiency onset after GC discontinuation is not uncommon and is sometimes unrecognized (e.g., in up to 4% of patients after nasal GC). There is no form of administration, dosing, treatment duration, or underlying disease that could exclude the possibility of CAI onset, although higher doses and longer use of GC result in the highest risk [7, 8]. Moreover, late CAI diagnosis is not uncommon and represents a frequent cause of hospitalization for hyponatremia, especially in the elderly [9, 10].

Apart from GC withdrawal, other conditions can induce adrenal insufficiency during treatment for COVID-19 or after severe acute respiratory syndrome coronavirus 2 (Sars-Cov-2) infection. Cortisol secretion may be insufficient in the event of critical illness-related corticosteroid insufficiency (CIRCI [11]). Moreover, the co-administration of certain antiretroviral drugs can trigger drug-drug interaction and enhance exposure to GCs metabolized through the CYP450 CYP3A pathway [12]. Coronavirus is able to exert a direct negative effect on pituitary corticotroph cells, which was also reported during the previous SARS outbreak in 2002–2003: 39.3% of patients developed transient hypocortisolism, in some cases with insufficient response to dynamic testing, who recovered 3–6 months later [13]. A group recently studied long-COVID patients, evaluated at least 3 months after the initial diagnosis: they reported reduced morning cortisol in 13% of cases, with adequate response to the stimulation test [14]. Moreover, primary adrenal injury consistent with bilateral adrenal hemorrhage has also been reported after COVID-19 [15].

Therefore, the development of adrenal insufficiency after COVID-19 is quite commonly encountered in clinical practice. We aimed to assess whether a predefined schedule to taper the high-dose and long-acting GC used in patients with COVID-19 can prevent adrenal crisis after GC discontinuation.

## Materials and methods

**Fig. 1:**
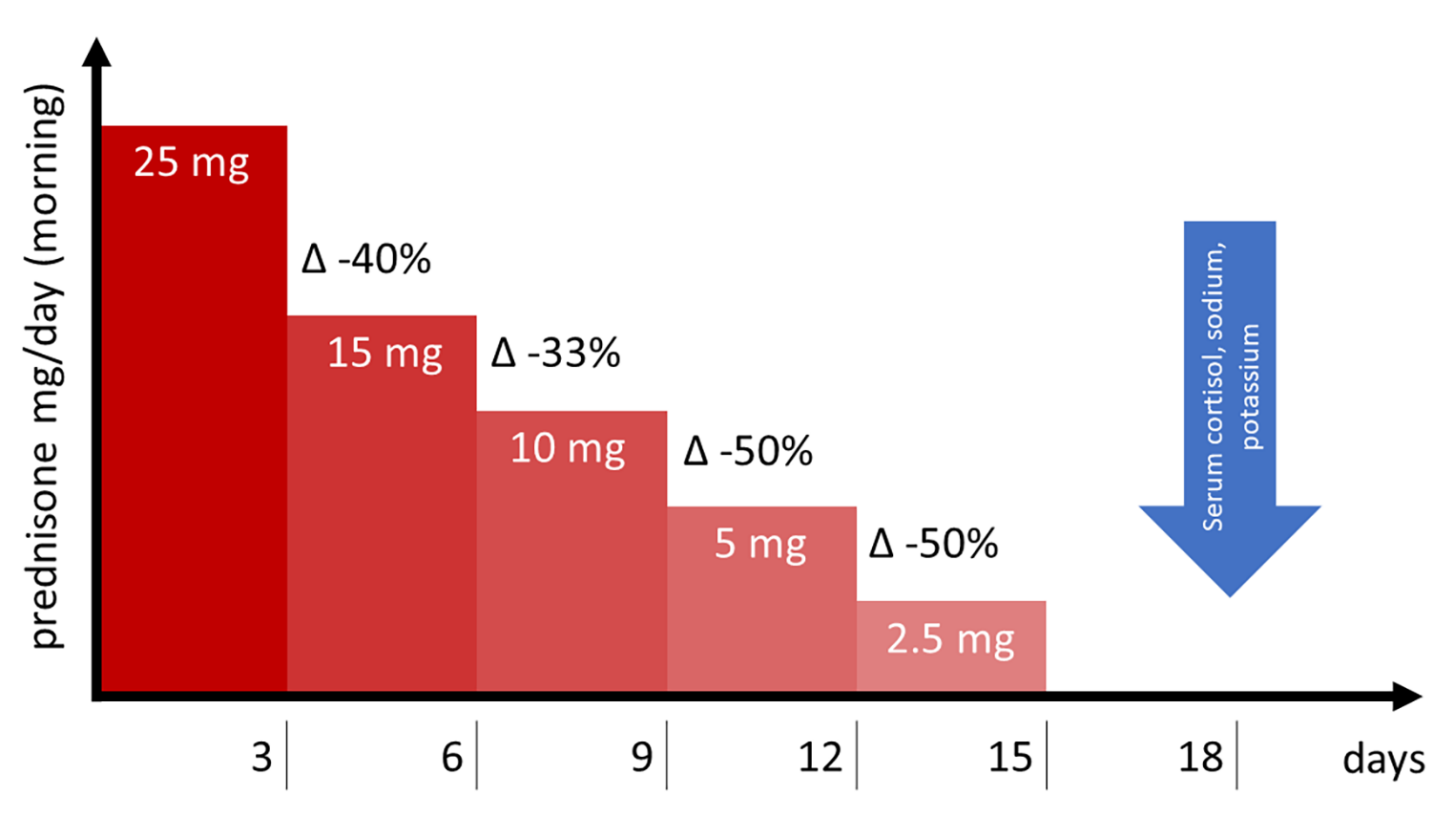
Glucocorticoid tapering regimen and morning serum sodium, potassium, and cortisol levels assessment

We performed an observational study from November 2021 to May 2022. Overall, 233 patients were admitted to the Geriatric Division of the University Hospital of Padova, Italy, with COVID-19 infection. Inclusion criteria were the following: severe COVID-19 infection (requiring high-flow oxygen, mechanical ventilation, or hospitalization in the intensive care unit [ICU]); treatment with dexamethasone 6 mg for 10 days, according to clinical condition; and GC tapering performed according to our schedule. The prescription for GC tapering was started the day after the last dexamethasone dose. It consists of a step-by-step GC tapering with prednisone, starting from 25 mg daily in the morning, followed by a 33–50% reduction every 3 days, until reaching 2.5 mg (Fig. 1). Morning serum sodium, potassium, and cortisol levels were assessed 3 days after the last dose of prednisone. There were 9 days of supraphysiological GC doses and 6 days of substitutive GC. Finally, 122 patients were selected for the study. If GC tapering started during hospitalization and the discontinuation occurred after discharge, a virtual consultation with the patient (or caregivers) was planned after the collection of a blood sample in order to assess the integrity of the HPA axis.

Patients with an early morning serum cortisol < 270 nmol/L (assessed 3 days after oral GC discontinuation, Fig. 1) or signs/symptoms of adrenal insufficiency onset were considered at risk of developing GC-induced CAI: substitutive GC treatment with cortisone acetate (25 mg divided into two doses, the higher administered in the morning) was started and an endocrinology outpatient visit was planned.

All selected patients gave their consent to the tapering schedule (when admitted to the Geriatric Division, after the *eventual* / final phase of hemodynamic instability); the study was conducted according to the principles of the Declaration of Helsinki. The Ethics Committee of the University Hospital of Padova approved the study and the virtual consultation (number 37,907 − 2020). Clinical data reported in eCRFs were collected anonymously. Continuous data were reported as median and interquartile range; categorical data were presented as absolute and relative (%) frequencies. The groups were compared with the chi-square test for categorical variables (the raw *P* values were adjusted with the Bonferroni method for multiple comparisons) and with the Mann-Whitney test for quantitative variables (after assessing the normality of distribution using the Kolmogorov–Smirnov Z test). The IBM SPSS v.24 software package for Windows (IBM Corp. Released 2016. IBM SPSS Statistics for Windows, Version 24.0. Armonk, NY, USA: IBM Corp.) was used to manage the database and perform the statistical analysis. The significance level was set at *p* < 0.05 for all tests.

## Results

The Geriatric Division of the University Hospital of Padova was devoted to COVID-19 patients from November 2021 to May to 2022, during the fifth pandemic wave. Overall, 233 patients were admitted (median age 82.5 years, female to male ratio 1.04:1, 74% completed the COVID-19 vaccination schedule, summarized in Table 1). A total of 24% were initially admitted to an ICU, 42% to the Infectious Disease Unit (and then moved to the Geriatric Division), and 26% were hospitalized after Emergency Department admission.

**Table 1:** Characteristics of the study population

Age at hospital admission (median and interquartile range)	82.5 (74–87)
Gender (female/overall, % female)	58/122 (47%)
Vaccination (yes/overall, % vaccinated)	85/122 (70%)
HFOT or NIV (yes/overall, % of HFOT or NIV)	43/122 (35%)
Remdesivir (yes/overall, % remdesivir)	75/122 (61%)
Monoclonal antibodies (yes/overall, % treatment)	35/122 (29%)

Data are presented as median (with interquartile range) or absolute numbers and frequencies

Abbreviations: HFOT: high-flow oxygen therapy, NIV: non-invasive ventilation

Among the entire cohort, 122 patients met the inclusion criteria. Female patients were 47% (58/122). The average hospital stay was 12 days; 70% of patients completed the vaccination schedule for SARS-CoV-2. During the hospitalization period, 43/122 patients (35%) needed high-flow oxygen therapy (HFOT) or non-invasive ventilation (NIV). Regarding antiviral medical therapies, 75/122 patients (61%) were treated with remdesivir and 35/122 patients (29%) with monoclonal antibodies for COVID-19, in addition to dexamethasone.

At the end of GC withdrawal, no adrenal crisis nor signs or symptoms of acute adrenal insufficiency were reported (summarized in Table 2). A morning serum cortisol level below the proposed threshold of 270 nmol/L was observed in two cases (respectively, 173 and 239 nmol/L), in whom a substitutive treatment with cortisone acetate was started. These two patients were reassessed with a low-dose short ACTH test (serum and salivary cortisol assessed before and 30 min after 1 µg of synacthen) 3 months after discharge: recovery of the HPA axis enabled discontinuation of the substitutive GC treatment. Mild hyponatremia (serum sodium 132 to 134 nmol/L) was observed in five patients: normal basal cortisol levels enabled exclusion of adrenal insufficiency-related hyponatremia in these patients. Median serum cortisol levels on the fourth day after prednisolone withdrawal, according to our schedule, was 427 nmol/L (interquartile range 395–479) in patients who were not treated with cortisone acetate. Likewise, electrolytes were normal after GC discontinuation (median sodium 140 nmol/L, interquartile range 138–142, median potassium 4 nmol/L, interquartile range 3.7–4.3).

**Table 2:** Descriptive analysis of patients with and without adrenal insufficiency

	Adrenal insufficiency (*n* = 2)	No adrenal insufficiency (*n* = 120)	*p* value
Monoclonal antibodies			0.507
Yes No	0	35 (30%)	
	2 (100%)	85 (70%)	
**Remdesivir**			0.146
Yes No	0	75 (62%)	
	2 (100%)	45 (38%)	
**HFOT/NIV**			0.417
Yes No	0	43 (36%)	
	2 (100%)	77 (64%)	
**Vaccination** Yes No			0.516
	1 (50%)	85 (70%)	
	1 (50%)	35 (30%)	
**Hospitalization** < 10 days 10–20 days > 20 days			0.158
	0	44 (37%)	
	1 (50%)	64 (53%)	
	1 (50%)	12 (10%)	

Data are presented as absolute numbers and frequencies. The groups were compared with the chi-square test for categorical variables

Abbreviations: HFOT: high-flow oxygen therapy, NIV: non-invasive ventilation

## Discussion

Corticosteroids are one of the mainstay treatments for *different* / numerous inflammatory conditions; however, their use is associated with a large number of side effects, among which GC-induced adrenal insufficiency is one of the most prominent / is prominent [16]. GC-induced adrenal insufficiency has to be considered in all patients during GC tapering, since all exogenous GCs exert negative feedback on the HPA axis with consequent inhibition of CRH-ACTH function, ultimately leading to pituitary corticotroph and adrenocortical cell atrophy. GC-induced adrenal insufficiency is a potentially life-threatening condition that requires a prompt diagnostic and therapeutic assessment; however, late diagnosis is not infrequent because GC-induced adrenal insufficiency presents with nonspecific signs or symptoms, such as fatigue and hyponatremia. A prompt and timely diagnosis is essential in order to plan optimal hormone replacement therapy [17, 18]. *The absence of predictive criteria for the development of adrenal insufficiency after GC withdrawal (treatment dose and duration, administration form, and scheduled cortisol measurements) is an important point to focus* / It is important to note that there are no predictive criteria for the development of adrenal insufficiency after GC withdrawal (treatment dose and duration, administration form, and scheduled cortisol measurements) [19, 20]. In 2015, Broersen et al. performed a systematic review and meta-analysis of the development of CAI after GC treatment. The prevalence of CAI varied from 4.2% for nasal corticosteroids to 52.2% for intra-articular GC formulations. Considering the underlying disease, 6.8% of asthma patients treated with inhalation GC and 60% of hematological patients developed CAI, respectively; considering the GC dose, 2.4% and 21.5% of patients developed CAI after GC low dose or high dose treatment, respectively. These findings highlight the fact that no dose, administration routes, underlying disease, or treatment duration of GC can safely reduce the likelihood of adrenal insufficiency [7]. Moreover, the cutoff to diagnose CAI should also be carefully considered. *In our study, the selected threshold for adrenal insufficiency (270 nmol/L) is indicated in the guidelines of the Society of Critical Care Medicine and the European Society of Intensive Care Medicine to define CIRCI* / In our study, the threshold for adrenal insufficiency (270 nmol/L) to diagnose CIRCI is recommended in the guidelines of the Society of Critical Care Medicine and the European Society of Intensive Care Medicine to define CIRCI [11].

Despite the widespread use of GCs, there are no clear data for guiding GC-induced adrenal insufficiency management, the studies being heterogeneous with a low level of evidence. However, it is known that patients who have been given systemic long-acting and high-potency GCs are at major risk of developing adrenal insufficiency compared with other conditions due to the GCs’ strong inhibitory effect on the HPA axis [21]. More specifically, daily systemic administration for at least 2–4 weeks of long-acting potent GC can lead to *strong* / critical adrenal suppression because of the continuous effect on the HPA axis. A higher GC dose also correlates *with stronger inhibition of* / with impaired CRH secretion. Furthermore, multiple daily doses and bedtime administration can also result in GC-induced development of adrenal insufficiency [22]. Indeed, evening GC administration affects clock-related genes in terms of genetic down-regulation due to higher GC sensitivity during the evening/night [23]. GC-induced adrenal insufficiency is not only a biochemical diagnosis but it is also of considerable clinical relevance, especially in cases of concomitant infections and of surgery which can precipitate adrenal insufficiency. In clinical practice, presentation of GC-induced adrenal insufficiency can be heterogeneous, ranging from asymptomatic disease to various degrees of cortisol deficiency and even causing adrenal crises [22]. The diagnosis of GC-induced adrenal insufficiency is a challenge since patients present with multiple non-specific symptoms that can be attributed to the underlying disease, for which GCs have been administered, or to CAI onset. Since mineralocorticoid secretion is preserved, symptoms are milder than in primary adrenal insufficiency: severe hemodynamic instability is infrequent and hyperkalemia is not a hallmark of CAI [24].

GC tapering is a crucial aspect *when dealing with* / in the design of a corticosteroid treatment scheme. The primary aim of any GC therapy is to prescribe the lowest effective dose for the shortest possible time. Despite there being until now no standardized GC tapering scheme, the differences in potency and pharmacokinetics of different types of GC must be taken into account, both with regard to HPA axis recovery and the effect of GC withdrawal syndrome (GWS) [25]. All patients should be advised as to the symptoms of GWS and provided with a plan to manage them; a useful recommendation is to increase GC to the most recent dose associated with well-being [22].

As demonstrated by our results, a planned GC tapering scheme shared by various specialists is able to prevent adrenal crises and to detect all CAI, even in the case of older patients whose condition is sometimes critical and who are hospitalized for an infectious condition. Moreover, it has been applied in a teleconsultation setting [26]. With the limitation of the number of patients who developed new-onset adrenal insufficiency, specific medical therapies (remdesivir and monoclonal antibodies) and the need for HFOT/NIV seemed not to increase the risk for GC-induced adrenal insufficiency. Otherwise, longer hospitalization might be suggested as a risk factor given that the two patients with CAI were discharged at least 10 days after admission.

Increased systemic cortisol availability is a vital component of the stress response during critical illnesses, severe stress, trauma, extensive surgery, or sepsis [27]. CIRCI is defined as a condition in which patients with prolonged critical illness require mechanical and/or pharmacological vital organ support for several days, with symptoms and signs compatible with adrenal insufficiency. CIRCI is thus an acquired adrenal insufficiency among the latter patients. Currently, there is no validated diagnostic test or imaging technique for the diagnosis [27]. In the case of suspicion of CIRCI and GC treatment, mainly with hydrocortisone, endocrinological follow-up is suggested for the patient. On the other hand, two studies have demonstrated that CIRCI is likely a reversible condition [28, 29]. In our experience, adequate follow-up of patients who developed central adrenal insufficiency, even in a mild form, after GC treatment is essential in order to better evaluate HPA axis integrity without interfering conditions related to concomitant drugs or even hospitalization-induced stress.

As limitations of the study we acknowledge the design (observational, not controlled or randomized), the low number of patients enrolled, and the limited number of patients who developed adrenal insufficiency after GC tapering. A defined follow-up protocol with control visits or examinations after COVID infection was scheduled only for selected patients (such as those discharged with residual respiratory symptoms, oxygen supplementation, or in the event of new-onset symptoms consistent with long-COVID disease after discharge). We selected a direct and not evidence-based arbitrary scheme; on the other hand, in the absence of a “gold-standard” or “evidence-based” schedule to taper glucocorticoid treatment, it did not appear safe for the patients to randomize them into different arms (with a blinded dose, different GCs, or placebo), with a cross-over arm designed either for low or high doses, which could develop new onset adrenal insufficiency or excess. The tapering scheme adopted is mostly based upon personal experience and clinical practice, shared with other non-endocrinologist physicians who use GCs in their clinical practice, and it can hence be used with frail patients in a non-endocrine setting (such as those in an internal geriatric ward).

To conclude, today, corticosteroids are widely used for their anti-inflammatory and immunosuppressive actions. Their use in the intensive care setting is frequently applied in life-threatening conditions, with concomitant use of other drugs. Therefore, optimal management of GC treatment and its discontinuation are crucial. Indeed, correct GC tapering plays a central role in ensuring treatment efficacy and must be shared among several physicians who take care of the patient at different stages of hospitalization and who are dealing with an evolving clinical condition. Our study has demonstrated that a schedule to taper GC treatment used in patients with severe COVID-19 can reduce the risk of adrenal crisis and acute adrenal insufficiency. Furthermore, our model of GC tapering can be offered for treatment of other medical conditions associated with frailty outside the setting in which it has been developed (e.g., a geriatric population hospitalized for COVID-19), including patients with chronic rheumatic, hematological, or respiratory diseases after GC treatment. Further studies are needed to establish whether our prednisolone tapering scheme can be used to wean patients off treatment after long-term GC therapy.
